# Transient Acidosis during Early Reperfusion Attenuates Myocardium Ischemia Reperfusion Injury via PI3k-Akt-eNOS Signaling Pathway

**DOI:** 10.1155/2013/126083

**Published:** 2013-11-07

**Authors:** Xin Qiao, Jinjin Xu, Qing-Jun Yang, Yun Du, Shaoqing Lei, Zhi-Hong Liu, Xinwei Liu, Huimin Liu

**Affiliations:** ^1^Department of Anesthesiology, Chongqing Zhoushan Hospital, Chongqing 404100, China; ^2^Department of Anesthesiology, The First Affiliated Hospital of Chongqing Medical University, Chongqing 404100, China; ^3^Department of Anesthesiology, Renmin Hospital of Wuhan University, Wuhan 430000, China; ^4^Department of Cardiac Surgery, Chongqing Zhoushan Hospital, Chongqing 404100, China; ^5^Department of Cardiac Surgery, Sun Yat-sen Cardiovascular Hospital, Shenzhen 515100, China

## Abstract

In this paper, we concluded that transient acidosis reperfusion conferred cardioprotection against myocardial ischemia reperfusion injury in isolated rat hearts through activating PI3K-Akt-eNOS pathway.

## 1. Introduction

During myocardial ischemia, tissue pH significantly declines and returns to normal after reperfusion [1]. Recently, studies reported that acidosis (pH < or = 7.0) protected profoundly against cell death during ischemia. However, the quick return from acidotic to normal pH after reperfusion may cause myocytes to lose viability. This worsening of postischemic injury is a “pH paradox” mediated by sudden or quick changes of intracellular pH (pHi) [2]. Normalization of pH after reperfusion initiates reactive oxygen species (ROS) formation and onset of the mitochondrial permeability transition pore (MPTP), which finally leads to cell death in cardiomyocytes, while acidosis can prevent mitochondrial permeability transition pore (MPTP) opening [3]. Therefore, prolongation of transient acidosis during early reperfusion may prevent the myocardium ischemia reperfusion injury.

Ischemic postconditioning, a novel strategy of cardioprotection consisting of the application of brief cycles of ischemia-reflow at the onset of reperfusion, represents a promising approach to protect the myocardium against ischemia and reperfusion injury [4, 5]. And this protection has been related to the activation of phosphatidylinositol 3-kinase-Akt dependent cytoprotective signaling pathway which is part of the reperfusion injury salvage kinase (RISK) that confers cardioprotection when activated at reperfusion [6, 7]. Additionally, Cohen et al. reported that ischemia postconditioning inhibits reoxygenated myocardium to produce reactive oxygen species and prevents MPTP formation by maintaining acidosis during the first 3 minutes of reperfusion [8]. Therefore, we hypothesized that direct acidotic infusion at the onset of reperfusion (acidosis postconditioning) could mimic ischemic postconditioning and induce the delayed recovery of pH and protect myocardium against ischemia reperfusion injury, and this protective effect may be mediated by PI3k-eNOS signaling pathway.

## 2. Methods

The experimental procedures conformed to the Guide for the Care and Use of Laboratory Animals published by National Institute of Health of the People's Republic of China and approved by the Institutional Animal Ethics Committee.

### 2.1. Isolated Perfused Rat Heart Preparation

Male Sprague-Dawley rats (450–550 g) were heparinised and then anaesthetized with urethan (700 mg/kg). The hearts were rapidly excised and mounted onto a Langendorff apparatus and perfused with modified Krebs-Henseleit bicarbonate buffer (KHB) that contained (in mM) 115.0 NaCl, 5.0 KCl, 1.2 MgSO_4_, 1.2 KH_2_PO_4_, 1.25 CaCl_2_, 25.0 NaHCO_3_, and 11.0 Glucose, equilibrated with 95% O_2_ and 5% CO_2_ to create perfusate pH 7.4, and maintained at 37°C as previously described [8]. Flow rate was initially adjusted to produce a perfusion pressure of 60 mmHg and was held constant thereafter. Then a water-filled latex balloon was inserted into left ventricle (LV) and inflated to obtain an end-diastolic pressure (LVEDP) between 6 and 8 mmHg. A pressure transducer connected to the perfusion line was used to continuously recorde the perfusion pressure.

### 2.2. Experimental Protocol

Rats were randomly divided into 7 groups (*n* = 12 per group). In control group, hearts were stabilized for 30 min and then subjected to no-flow global ischemia by inflation of the coronary balloon for 30 min followed by 120 min of reperfusion with normal KHB (C group). Postconditioning was achieved by 6 cycles of 15 s reperfusion and 15 s occlusion after no-flow global ischemia (IPO group). In the acidotic reperfusion group, hearts were perfused with KHB adjusted at pH 6.9 for the first 3 min of reperfusion (H^+^ group). The acidotic perfusion buffer was adjusted by equilibrating with 80% O_2_/20% CO_2_ (pH 6.9). To test whether alkali abolished the protection of ischemia postconditioning, hearts were postconditioned with alkalotic buffer which was equilibrated with 100% O_2_ (pH 7.8) (IPO + OH^−^). In OH^−^ group, hearts were only reperfused with alkalotic buffer without ischemia postconditioning. Finally, PI3k specific inhibitor wortmannin (100 nmol/L) was added to the acidotic perfused and infused into heart for the first 3 min of reperfusion after ischemia (H^+^ + wort group). In wort group, hearts were reperfused with normal KHB cotreatment of wort (100 nmol/L). After the first 3 min of reperfusion with respective treatment, all groups were switched to buffer equilibrated with 5% CO_2_. In all hearts, reperfusion lasted for 120 min.

### 2.3. Infarct Size Measurement

After 120 min of reperfusion, the coronary artery was reoccluded. The hearts were weighed and frozen at −20°C for 20 min and then underwent horizontal long axis slicing at a thickness of 1-2 mm. The slices were incubated for 15 minutes at 37°C in buffered 1% triphenyltetrazolium chloride in order to stain noninfarcted myocardium brick red. Slices were then fixed in 10% formalin for 5 minutes. The infarct size was defined as the ratio of the weight of the necrotic zone to that of the ischemic zone as previously described [9].

### 2.4. Quantification of Nitric Oxide Release in Isolate Hearts

NO release was measured in the perfusates previously described [10] under ultraviolet/visual spectrometry using an extinction coefficient of 411 nm to 401 nm at ambient temperature. The respective measurements were performed using a double-beam spectrometer (DW2000, SLM-Aminco, USA).

### 2.5. Western Blot Assay for Akt and eNOS

Frozen heart tissues were homogenized using lysis buffer (20 mmol/L Tris-HCl, PH 7.5, 50 mmol/L 2-mercaptoethanol, 5 mmol/L EGTA, 2 mmol/L EDTA, 1% NP40, 0.1% sodium dodecyl sulfate (SDS), 0.5% deoxycholic acid, 10 mmol/L NaF, 1 mmol/L PMSF, 25 mg/mL leupeptin, and 2 mg/mL aprotinin) for 30 min then sonicated and centrifuged at 12000 g for 20 min at 4°C. Protein concentrations were determined using the Bradford assay (Bio-Rad, USA). Samples containing equal amounts were separated on a 10% SDS-polyacrylamide gel, and then proteins were transferred to PVDF membrane overnight at 4°C. Membranes were blocked with 5% nonfat milk in Tris-buffered saline (TBS)-Tween for 1 hr and were incubated with anti-Akt or anti-eNOS antibodies and GAPDH (Cell Signaling Technology, Beverly, MA) at 1 : 1000 dilution overnight at 4°C. After washing with phosphate buffered saline-Tween (PBST) three times for 30 min, membranes were then incubated with horseradish peroxidase (HRP)-conjugated anti-rabbit IgG at 1 : 2000 dilution for 1 h. Protein bands were developed with enzymatic chemiluminescence, and images were measured by a densitometer with analysis software.

### 2.6. Measurement of Free 15-F2t-Isoprostane

Free 15-F2t-isoprostane (15-F2t-IsoP), a specific marker of oxidative stress, was measured by using an enzyme-linked immunoassay kit (Cayman chemical, Ann Arbor, MI) as described [11]. Perfusate and homogenized heart tissue (in PBS) were purified using Affinity Sorbent and Affinity Column (Cayman chemical, Ann Arbor, MI) then processed for analysis, according to the protocol provided by the manufacturer. The values of plasma or cardiac free 15-F2t-IsoP were expressed as pg/mL in perfusate or pg/mg protein in cardiac homogenates, respectively.

### 2.7. Statistical Analysis

Data are presented as means ± standard error of the mean (S.E.M.). Data were analysed by the ANOVA within the same group and between groups. Multiple comparisons of group means were analyzed by Tukey's test. The analysis was performed using statistical software package (GraphPad Prism, San Diego, CA, USA). Significant difference was defined as *P* ≤ 0.05.

## 3. Results

### 3.1. Cardiac Function

As shown in Table 1, none of the baseline data before ischemia represented statistically significant difference among all groups. In control hearts subjected to 30 min ischemia and 30 min reperfusion, LVEDP was higher and both +dp/dt_max⁡_ and −dp/dt_max⁡_ were lower compared with its baseline data before ischemia. IPO treatment significantly decreased LVEDP and increased +dp/dt_max⁡_ and −dp/dt_max⁡_ at the time of reperfusion (30 min) as compared with the I/R group (*P* < 0.05, versus I/R). When postconditioning was performed with alkalotic perfusate, it was no longer protective. Acidic reperfusion (H^+^ group) also improved cardiac function, similar to the effect of postconditioning. However, this protection was reverted by PI3k inhibitor wort (*P* < 0.05) (Table 1) although wortmannin alone in normal KHB perfusate did not significantly alter cardiac function compared with control group (*P* > 0.05).

### 3.2. Infarct Size

**Figure 1:**
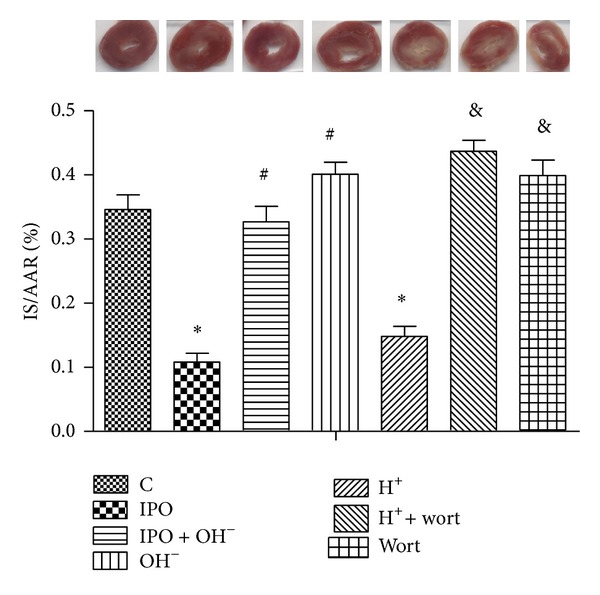
Infarct size expressed as a percentage of risk area. *P < 0.05 versus C; ^#^ *P* < 0.05 IPO + OH^−^ or OH^−^ versus IPO; ^&^ *P* < 0.05 H^+^ + wort or wort versus H^+^.

Postconditioning decreased infarct size from 34.6% ± 2.3% of risk zone in control hearts to 10.8% ± 1.4% (*P* < 0.01) (Figure 1). However, this protection was abolished by alkalization perfusate of postconditioning (*P* < 0.05) (Figure 1). However, alkalotic buffer reperfusion alone further increased infarct size compared with control group (*P* < 0.05) (Figure 1). Acidic perfusate (H^+^ group) mimicked the protection of postconditioning, which was reverted by PI3k inhibitor wortmannin (*P* < 0.05) (Figure 1) Furthermore, wortmannin alone in normal KHB perfusate increased the infarct size compared with control hearts (*P* < 0.05).

### 3.3. Nitric Oxide Release

**Figure 2:**
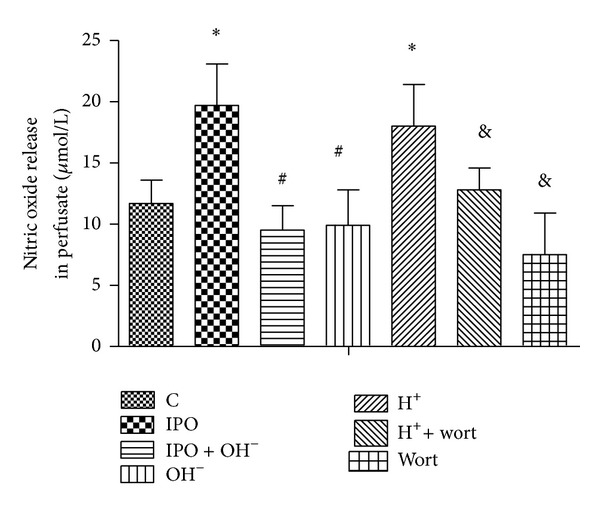
Effects of postconditioning and acidosis perfusate on the nitric oxide release in perfusate. *P < 0.05 versus C; ^#^ *P* < 0.05 IPO + OH^−^ or OH^−^ versus IPO; ^&^ *P* < 0.05 H^+^ + wort or wort versus H^+^.

Postconditioning significantly increased nitric oxide release at the time point of reperfusion (30 minutes) compared with that of control group (*P* < 0.05) (Figure 2), which was reverted by alkalized perfusate of postconditioning. Acidotic perfusion buffer also increased the level of nitric oxide, which was blocked by cotreatment of PI3k inhibitor wortmannin. While level of nitric oxide in wort group was even more lower compared with control group (*P* < 0.05), this indicated that the dosage of wortmannin used in this study was enough to inhibit PI3k activation.

### 3.4. Protein Expression of Akt and eNOS

**Figure 3:**
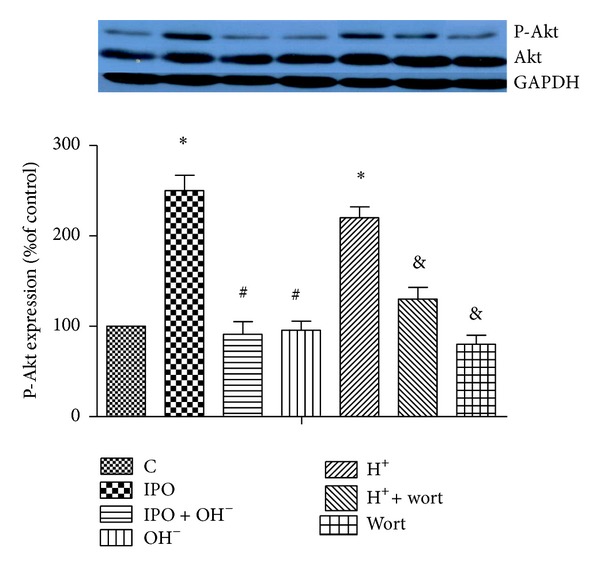
Effects of postconditioning and acidosis perfusate on the activation of Akt in ischemia myocardium. *n* = 12 for each. *P < 0.05 versus C; ^#^ *P* < 0.05, IPO + OH^−^ or OH^−^ versus IPO; ^&^ *P* < 0.05, H^+^ + wort or wort versus H^+^.

**Figure 4:**
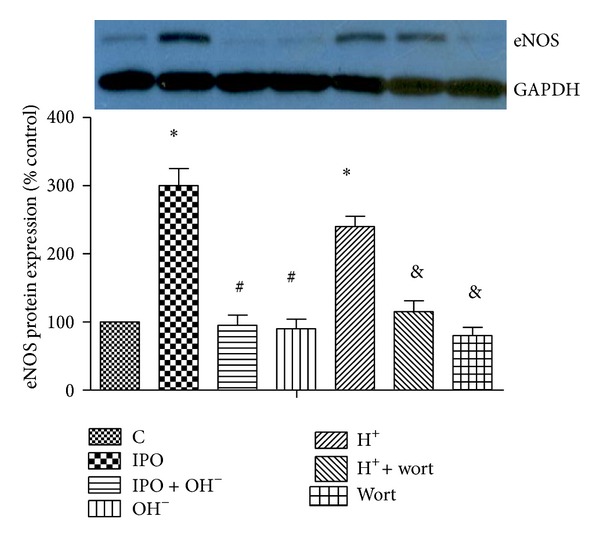
Effects of postconditioning and acidosis perfusate on the protein expression of eNOS. *P < 0.05 versus C; ^#^ *P* < 0.05, IPO + OH^−^ or OH^−^ versus IPO; ^&^ *P* < 0.05, H^+^ + wort or wort versus H^+^.

As shown in Figures 3 and 4, postconditioning induced the phosphorylation of Akt and eNOS protein expression compared with that of control. However, cotreatment with alkalotic perfusate abolished these effects. Acidotic perfusion buffer copied the effect of postconditioning, evidenced as increased phosphorylation of Akt and eNOS protein expression. However, this effect was reverted by cotreatment of PI3k inhibitor wortmannin. Infusion of wortmannin alone also inhibited the phosphorylation of Akt and eNOS expression.

### 3.5. Level of Perfusate and Tissue Free *15-F2t-Isoprostane*

Compared with the control group, the levels of free 15-F2t-IsoP were significantly decreased in both the perfusate and heart tissues in IPO group (*P* < 0.01 or *P* < 0.05 versus C, Table 2). Postconditioning with alkaline perfusate increased perfusate and heart tissue 15-F2t-IsoP to a level comparable to that in the control (*P* < 0.05, IPO + OH^−^ versus PO; *P* > 0.05, IPO + OH^−^ versus control, Table 2). Acidotic perfusion buffer decreased both the perfusate and cardiac tissues of free *15-F2t-isoprostane*, which was reverted by wortmannin. Wortmannin alone did not affect level of both perfusate and tissue free *15-F2t-isoprostane *compared with control group.

## 4. Discussion

This study demonstrated, in the isolated rat heart, that ischemia postconditioning exerted cardioprotection by delaying pHi recovery, since this effect was abrogated by alkaline perfusate. Acidic perfusion mimicked the cardioprotective effect of postconditioning to limit myocardial infarct size, improve myocardial function, and inhibit ROS production by prolongation of intracellular acidosis during reperfusion. Furthermore, acidic perfusion increased Akt phosphorylation, eNOS expression, and nitric oxide release, which were reverted by cotreatment with the PI3k inhibitor wortmannin. These results suggested that acidic perfusion exerts cardioprotection by activating PI3k-Akt-eNOS signaling pathway during the early reperfusion phase.

Combined action of different transport systems, including Na^+^/H^+^-exchanger, Na^+^/HCO_3_^−^ symport, and H^+^-coupled lactate efflux, plays a crucial role in the recovery of pHi during reperfusion. Washout of lactate, H^+^, and CO_2_ results in normalization of pHi [12]. Therefore, reducing catabolite washout can attenuate transmembrane H^+^ gradient and decrease the activity of Na^+^/H^+^-exchanger and Na^+^/HCO_3_^−^ symport. Intracellular low pHi inhibits Ca^2+^-dependent hypercontracture and opening of MPTP during initial reperfusion which protect the heart from ischemia reperfusion injury. Javier et al. [13] found that acidic reperfusion can prolong intracellular acidosis, but it lasts only for the first 3 minutes of reperfusion. Exceeding this time hearts rapidly restored pHi despite extracellular acidosis. In our study, we performed perfusion with acidic buffer during the first 3 minutes of reperfusion, and our results also found that 3 minutes of acidic perfusion mimicked those protective effects of ischemia postconditioning. However, the underlying mechanism is not well understood.

A variety of studies have demonstrated that postconditioning procedures protect the heart against reperfusion injury by activating RISK pathways at the time of reperfusion through the phosphorylation of PI3k/Akt pathways. In the current study, we have shown that postconditioning increased activation of PI3k/Akt and limited myocardial infarct size, while infusion of alkaline perfusate abolished the phosphorylation of Akt induced by postconditioning and blunted the cardioprotection of postconditioning, which is consistent with Fujita et al.'s report [14]. Our data further showed that acidic perfusion mimicked the protective effects of ischemia postconditioning, evidenced as decreased infarct size and improved cardiac function, accompanied with increased activation of Akt. However, PI3k inhibitor wortmannin reverted the protection of acidic perfusate and decreased phosphorylation of Akt. These results indicated that acidosis reperfusion protected the myocardium from I/R injury at least in part through the activation of PI3k-Akt pathway.

Furthermore, the activation of PI3k/Akt induced by ischemia postconditioning further activates downstream targets such as endothelial nitric oxide synthase (eNOS) which will induce the release of NO [15–17]. NO can lead to the inhibition of the mitochondrial permeability transition pore opening which may be a key end-effector of cell death and cardioprotection [18, 19]. Studies found that inhibition of NO synthase blunted the infarct size-limiting effects of postconditioning [20]. In the current study, we have shown that acidosis reperfusion increased NO release in the perfusate and enhanced myocardial eNOS protein expression, while infusion of alkaline perfusate reduced the level of NO in perfusate as well as myocardial eNOS protein expression, accompanied with decreased activation of Akt. Besides these, PI3k inhibitor wortmannin reduced NO release and eNOS protein expression, paralleled with decreased phosphorylation of Akt and eNOS. All these results indicated that acidosis induced the activation of PI3k-Akt-eNOS pathway whereby it increased NO release and thus led to the attenuation of ischemia reperfusion injury.

Additionally, restoration of oxygenation during brief reperfusion causes mitochondria to produce ROS which could induce oxidative stress and subsequently lead to the myocardial damage during reperfusion, while acidosis during reperfusion has been reported to attenuate ROS generation [3]. Administration of ROS scavenger alleviated myocardial ischemia reperfusion injury [21, 22]. In our study, we found that acidic perfusate reduced the levels of both perfusate and heart tissue 15-F2t-isoprostane, a specific marker of oxidative stress, similar to the effect of postconditioning. It suggested that acidotic perfusate could inhibit oxidative stress caused by myocardial ischemic reperfusion injury. However, this effect of acidotic perfusate was blocked by cotreatment with the PI3k inhibitor wortmannin. These findings collectively indicate that acidosis attenuated ischemia reperfusion injury partly through activating PI3k signaling pathway to inhibit oxidative stress.

In summary, acidic reperfusion exactly mimics the protection of postconditioning. Acidosis confers cadioprotection by inhibiting ROS production and increasing NO release through activating PI3k-Akt-eNOS pathway.

## Figures and Tables

**Table 1:** Cardiac function for experimental groups (*n* = 10, $\bar{x}\pm s$).

Group	LVEDP (mmHg)		+*dp*/*dt*_max⁡_ (mmHg/s)		−*dp*/*dt*_max⁡_ (mmHg/s)	
	Baseline	30 min reperfusion	Baseline	30 min reperfusion	Baseline	30 min reperfusion
Control	5.06 ± 1.02	56.98 ± 9.46	2703 ± 135	2051 ± 81	2637 ± 124	2004 ± 67
IPO	7.25 ± 1.17	43.18 ± 4.62*	2811 ± 116	2297 ± 82*	2798 ± 136	2282 ± 87*
IPO + OH^−^	6.16 ± 1.29	48.45 ± 7.15^∗#^	2768 ± 222	2057 ± 246^#^	2686 ± 149	1996 ± 296^#^
OH^−^	6.52 ± 1.21	56.46 ± 5.29	2759 ± 237	2121 ± 248	2690 ± 240	2101 ± 243
H^+^	7.19 ± 0.75	36.34 ± 6.30*	2803 ± 179	2274 ± 135*	2801 ± 171	2250 ± 148*
H^+^+ wort	6.55 ± 1.41	51.56 ± 5.43^&^	2846 ± 148	2199 ± 140^&^	2802 ± 142	2070 ± 134^&^
Wort	6.92 ± 1.26	53.55 ± 5.77	2734 ± 187	1913 ± 82	2699 ± 192	1898 ± 78

*P < 0.05 or 0.01 versus control group; ^#^ *P* < 0.05, IPO + OH^−^ versus IPO; ^&^ *P* < 0.05, H^+^ + wort versus H^+^.

**Table 2:** Level of perfusate and tissue free *15-F2t-isoprostane*.

	C	IPO	IPO + OH^−^	OH^−^	H^+^	H^+^ + wort	Wort
Perfusate (pg/mL)	303.1 ± 15.9	225.7 ± 11.4*	311.5 ± 18.6^#^	312.5 ± 11.7	235.5 ± 10.6*	308 ± 11.6^&^	310.3 ± 10.0
Heart tissue (pg/mg protein)	255.3 ± 13.7	208.1 ± 17.1*	262.1 ± 12.1^#^	270.2 ± 10.3	210.7 ± 12.7*	211.1 ± 13.9^&^	265.1 ± 153

*P < 0.05 or 0.01 versus control group; ^#^ *P* < 0.05, IPO + OH^−^ versus IPO; ^&^ *P* < 0.05, H^+^ + wort versus H^+^.
